# Combined Phacoemulsification and Goniosynechialysis under an Endoscope for Chronic Primary Angle-Closure Glaucoma

**DOI:** 10.1155/2018/8160184

**Published:** 2018-02-08

**Authors:** Li Nie, Weihua Pan, Aiwu Fang, Zhangliang Li, Zhenbin Qian, Lin Fu, Yau Kei Chan

**Affiliations:** ^1^School of Ophthalmology and Optometry, Eye Hospital, Wenzhou Medical University, Wenzhou, Zhejiang, China; ^2^Department of Mechanical Engineering, University of Hong Kong, Pokfulam, Hong Kong

## Abstract

**Purpose:**

To investigate the clinical efficacy and safety of combined phacoemulsification with goniosynechialysis (GSL) under an ophthalmic endoscope for chronic primary angle-closure glaucoma and coexisting cataract.

**Methods:**

This is a retrospective study. The intraocular pressure (IOP), best-corrected visual acuity (BCVA), and number of glaucoma medications at baseline and each postoperative follow-up visit were recorded. Other measurements included supraciliochoroidal fluid measured by anterior segment optical coherence tomography, corneal endothelial cell density (ECD), and peripheral anterior synechia (PAS). All patients were followed for more than a year.

**Results:**

Thirty-eight eyes of 31 patients were included. The mean follow-up duration was 16.3 ± 3.9 months. The IOP decreased from 22.2 ± 9.3 mmHg at baseline to 15.4 ± 4.2 mmHg at the last follow-up (*P* < 0.001). The mean number of glaucoma medications (0.1 ± 0.6) at the last follow-up was significantly lower than the preoperative number (2.3 ± 1.1) (*P* < 0.001). All patients achieved improved or stable visual acuity after surgery. All patients achieved a complete opened angle after GSL. The postoperative complications included hyphema (7.9%), exudation (5.3%), transiently elevated IOP (55.3%), and supraciliochoroidal fluid (40%).

**Conclusions:**

Combined phacoemulsification and GSL under an endoscope can completely reopen PAS and is an effective and safe method for patients with chronic primary angle-closure glaucoma and coexisting cataract.

## 1. Introduction

Primary angle-closure glaucoma (PACG) is a disease initiated by angle closure, which leads to an elevated intraocular pressure (IOP) and causes subsequent optic nerve damage. There are multiple reasons which lead to angle closure in PACG patients, such as pupillary block, plateau iris, and lens-related factors [1, 2].

Filtering surgery is currently the most common treatment for PACG patients. However, a high incidence of postoperative complications, including but not limited to shallow anterior chamber, macular edema induced by low IOP, choroidal effusion, thin-walled bleb, and endophthalmitis caused by bleb leakage, was observed [3]. In addition, long-term postoperative bleb scarring significantly reduces the success rate of filtering surgery [4].

To some PACG patients, goniosynechialysis (GSL) is a safe and effective surgical choice [5]. GSL can separate peripheral anterior synechia (PAS) from the angle, expose the functional trabecular meshwork, and therefore restore its filtering function [2, 6]. For patients with cataract and PACG, combined phacoemulsification with GSL can reduce IOP and improve visual acuity at the same time [5]. Combined phacoemulsification and GSL (phaco-GSL) leads to a deepened anterior chamber, reopened angle, and resolution of a pupillary block caused by the lens. This results to a reduction of IOP. The success rate of phaco-GSL is reported to be 80%–100% [2, 6, 7]. The lens removal can relieve the pupillary block with other lens-related factors and eliminate the anteriorly positioned ciliary body, thus reducing the risk of angle closure [8]. If GSL was not combined with cataract surgery, a long-term IOP-lowering effect is difficult to achieve because the pupillary block and other lens-related mechanisms were not fundamentally resolved [9, 10]. The combination of cataract extraction and GSL synergistically reduces the IOP. GSL is usually performed with the assistance of a gonioscope. However, gonioscope-assisted GSL requires either a microscope or an eyeball to be tilted, and the quality of the image is affected by corneal edema. The ophthalmic endoscope provides a direct view of the PAS and trabecular meshwork [11].

In this study, we retrospectively investigated the clinical efficacy and safety of combined phacoemulsification and GSL assisted by an ophthalmic endoscope (phaco-OE-GSL) for chronic PACG patients with cataract. We used an ophthalmic endoscopic system to assist in the procedure of GSL. Under the ophthalmic endoscopic system, the surgeon can directly observe the angle structure and relative anatomical positions. Hence, PAS can be separated between the iris and the trabecular meshwork under direct view [11].

## 2. Patients and Methods

### 2.1. Patients

This research was approved by the Ethics Committee of Wenzhou Medical University. Patients with chronic PACG and cataract who underwent phaco-OE-GSL at the Eye Hospital of Wenzhou Medical University from July 2014 to April 2015 were included. They were followed for at least 1 year. The diagnosis of chronic PACG was based on the diagnostic criteria of the International Society of Geographic and Epidemiologic Ophthalmology [12]. Inclusion criteria were chronic PACG patients with various degrees of cataract that had reduced visual function, an angle closure of more than 90 degrees, and an IOP higher than 21 mmHg with glaucomatous optic nerve damage. Exclusion criteria included secondary angle-closure glaucoma or those who had undergone ophthalmic surgeries other than laser peripheral iridotomy. All patients underwent GSL directed by an ophthalmic endoscope after phacoemulsification and intraocular lens implantation.

### 2.2. Main Measurements

The preoperative measurements included best-corrected visual acuity (BCVA) using LogMAR chart records, slit lamp and fundus examination, IOP measurement using Goldmann applanation tonometry, the extent of PAS recorded via gonioscopy, axial length measurement using IOLMaster (Carl Zeiss Meditec; Germany), and corneal endothelial cell density (ECD) by noncontact specular microscopy (Tomey EM-3000, Nagoya, Japan). The equipment included a URAM E2 laser endoscopic system (Endo Optiks), a light source, and a video recording system attached to a 23G probe.

The BCVA, IOP, and number of glaucoma medications and complications were recorded at 1 week and 1, 3, 6, 12, and 18 months postoperatively and at the last follow-up examination. The supraciliochoroidal fluid was investigated through anterior segment optical coherence tomography (AS-OCT; ZEISS; Germany) 1–3 days postoperatively in some patients. Patients were examined at the 0-, 90-, 180-, and 270-degree meridians of the anterior segment. A part of the ciliary body was also captured during imaging. The grading of the supraciliochoroidal fluid conformed to the following: grade I, <1/2 ciliary body thickness; grade II, 1/2–1 ciliary body thickness; and grade III, >1 ciliary body thickness [13]. Noncontact specular microscope counting ECD was examined at 1–3 months postoperatively. PAS recurrence was examined via gonioscopy after 6 months postoperatively. A complete success was defined as an IOP between 6 and 21 mmHg without glaucoma medications and additional surgery.

### 2.3. Surgical Procedure

**Figure 1:**
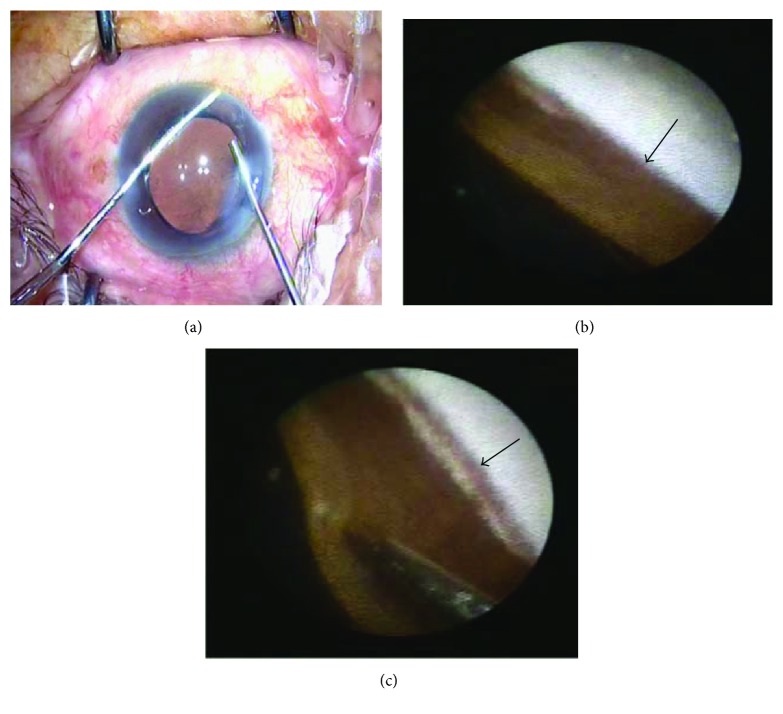
GSL under the ophthalmic endoscope. (a) The endoscope probe was inserted into the anterior chamber from the main incision, and GSL was performed using a modified iris repositor. (b) PAS (arrow) was observed under the endoscope before the mechanical GSL. (c) The iris repositor pressed down the iris foot to separate the PAS under the endoscope. The pigments were visible after exposing the trabecular meshwork (arrow).

Topical anesthesia (0.5% proparacaine HCl) was applied 10 mins before surgery. Clear corneal phacoemulsification was performed through a 2.2 mm main incision and 1 mm lateral incision. The subsequent procedures included in the order of continuous curvilinear capsulorhexis, phacoemulsification, removal of the residual cortex, and implantation of a foldable intraocular lens in the capsular bag. A viscoelastic agent was injected into the anterior chamber to press down the iris foot of the appositional PAS (visco-GLS). The ophthalmic endoscopic probe was then inserted into the anterior chamber to investigate the angle. If residual PAS was found, further mechanical GSL was performed using a modified iris repositor to separate PAS until the trabecular meshwork was observed (Figure 1). In all cases, we used a 23G endoscopic probe with a diameter of 0.6 mm to enter the anterior chamber through the main incision. When necessary, we expanded the side incision and allowed the probe to enter the anterior chamber for either observation or separation. After GSL, the viscoelastic agent was replaced with Ringer's solution and the incision was sealed by corneal stromal hydration or closed using a 10-0 nylon suture. Subconjunctival injection of dexamethasone was administered in some patients. All surgical procedures were performed by the same surgeon (Dr. Weihua Pan).

Postoperatively, all patients were prescribed topical use of tobramycin and dexamethasone eyedrops for 4 weeks (4×/d), nonsteroidal anti-inflammatory eyedrops for 4 weeks (4×/d), and 0.5% pilocarpine eyedrops for 4 weeks (2×/d).

### 2.4. Statistical Analysis

Data are presented as mean with standard deviation. BCVA and the number of glaucoma medications were compared using the Wilcoxon signed-rank test. A comparison of the extent of preoperative and postoperative ECD was performed using the paired *t*-test. Repetitive measures analysis of variance was used to compare the preoperative and postoperative IOPs. We use the Statistical Package for the Social Sciences, version 20 (SPSS, Chicago, IL, USA), to perform the above-mentioned analyses.

## 3. Results

A total of 31 patients (38 eyes) were recruited in this study. The mean age was 68.3 ± 11.1 years (range, 43–85 y), and 6 of them were men. Laser peripheral iridotomy had been performed in 31 eyes before surgery. The mean follow-up time was 16.3 ± 3.9 months (range, 13–23 mo).

**Figure 2:**
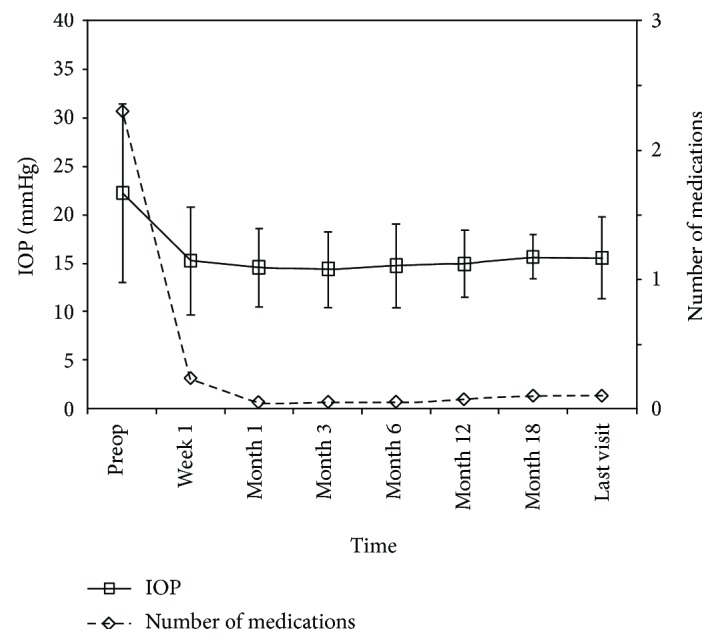
The IOP and mean number of glaucoma medications at baseline and different time points postoperatively.

The mean preoperative IOP under medication therapy was 22.2 ± 9.3 mmHg (range, 7–45 mmHg). The postoperative IOPs were as follows (Figure 2): at 1 week, 15.1 ± 5.5 mmHg; 1 mo, 14.4 ± 3.9 mmHg; 3 mo, 14.3 ± 3.8 mmHg; 6 mo, 14.8 ± 4.3 mmHg; 12 mo, 14.8 ± 3.3 mmHg; 18 mo, 15.6 ± 2.1 mmHg; and last follow-up, 15.4 ± 4.2 mmHg. At each time point, the postoperative IOP was significantly lower than that at baseline (*P* < 0.001). No postoperative hypotony was observed (i.e., ≤5 mmHg at 2 consecutive follow-up examinations).

The number of medications was significantly lower compared with that at baseline at all time points after surgery (*P* < 0.001) (Figure 2). The mean preoperative number of glaucoma medications was 2.3 ± 1.1 (range, 0–4), while it was 0.1 ± 0.6 at the last follow-up, with one patient using 4 eyedrops on one eye. At the last follow-up, 37 operated eyes attained a controlled IOP lower than 21 mmHg without glaucoma medications, and the complete success rate was 97.4%.

**Figure 3:**
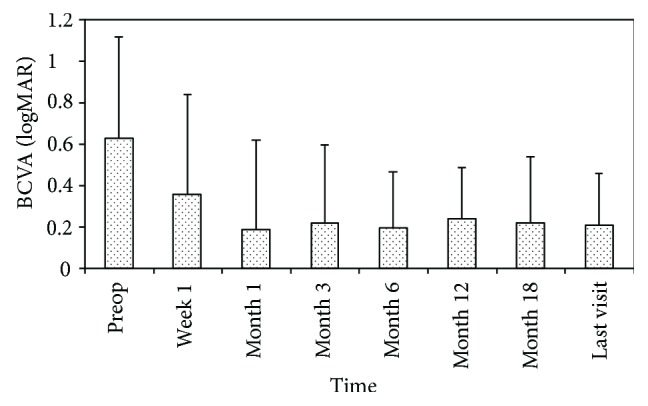
LogMAR best-corrected visual acuity at baseline and different time points postoperatively.

Postoperative BCVA was significantly improved compared with that at baseline at all postoperative time points (*P* = 0.004) (Figure 3). The visual acuity was 0.63 ± 0.49 at baseline and 0.21 ± 0.25 at the last follow-up (*P* = 0.007). Thirty-two eyes achieved improved visual acuity (84.2%), and 6 eyes attained stable visual acuity (15.8%).

The preoperative PAS range was between 90 and 360° with an average range of 184.7 ± 99.3°. The angle was completely opened in 9 eyes (23.7%), and the residual PAS range was 102.9 ± 97.5° after visco-GSL. In such cases, the residual PAS was handled by a mechanical separation to achieve a completely opened angle. The mean postoperative gonioscopic time was 12.6 ± 5.3 months (range, 8–16 months). 68.4% (26/38) eyes suffered various degrees of postoperative PAS recurrence, with a recurrence range between 15 and 270°. We observed different degrees of pigmentation located in the trabecular meshwork of the opened angle in some of the operated eyes.

One eye suffered from poorly controlled IOP, and four kinds of glaucoma medications were applied after GSL. We performed glaucoma valve implantation at 6 months postoperatively. The glaucoma history of the patient was 84 months, with preoperative 180° PAS, anterior chamber depth of 2.9 mm, axial length of 24.2 mm, and C/D of 1.0. The postoperative PAS was 90°, with grade III pigment accumulation in the trabecular meshwork (pigment grading method referring to the Scheie sorting technique) [14].

The corneal ECD was decreased from 2667.15 ± 320.80 preoperatively to 2359.29 ± 387.51 at 1–3 months postoperatively (24 eyes of 20 patients, *P* = 0.004). In terms of percentage, ECD was decreased by 11.54% compared with baseline.

Intraoperative complications included hyphema in 5 eyes, which was controlled after oppression hemostasis by a viscoelastic agent, and aqueous reflux in one eye, which underwent vitreous puncture and intravenous injection of mannitol to deepen the anterior chamber. All these patients successfully underwent GSL after the treatment of the above-mentioned complications. The postoperative complications included hyphema in 3 eyes (7.9%), transiently elevated IOP in 21 eyes (55.3%), and grade I supraciliochoroidal fluid in 6 eyes (40% in 15 eyes). Two eyes (5.3%) had anterior chamber exudation, which was absorbed within one week after conservative treatment. The transiently elevated IOP had lasted for 2.7 ± 4.3 days (range 2 h–20 d) and was controlled after topical application of glaucoma medications or anterior chamber paracentesis. Supraciliochoroidal fluid recovered within 3 weeks after conservative treatment. There were no other complications observed, such as iridodialysis, shallow anterior chamber, or ciliary block glaucoma.

## 4. Discussion

In this retrospective study, we analyzed the clinical efficacy and safety of phaco-OE-GSL for chronic PACG patients with cataract. Overall, patients achieved controlled postoperative IOP, improved or stable postoperative visual acuity, and reduced number of glaucoma medications. Moreover, the range of postoperative PAS decreased, and no serious perioperative complications were observed. The complete success rate was 97.4% at the last follow-up. These indicate that phaco-OE-GSL is effective and safe for patients of chronic PACG with various degrees of cataract.

In our previous study, we reported that phaco-OE-GSL was viable for PACG patients with an acute attack [11]. However, in that study, only 12 patients were included, and the mean follow-up period was only 7 months. Moreover, only a few subsequent studies concerning GSL under an endoscope were performed. Maeda et al. [15] retrospectively reviewed 13 PACG patients who underwent GSL under an endoscope with a success rate (IOP < 21 mmHg without medications) of 100%, after an average follow-up of 11 months. They also used a 23G endoscopic probe to achieve 360° complete GSL. However, most of their cases were acute PACG and received phacoemulsification combined with GSL without IOL implantation. In contrast, we performed IOL implantation together with GSL. Also, 21% eyes in their study underwent laser gonioplasty after surgery and had no investigation of the PAS recurrence due to lack of enough postoperative gonioscopic data. These justify the importance of our study.

Teekhasaenee and Ritch [2] reported a success rate (IOP < 20 mmHg without medications) of 90.4% for combined phacoemulsification and GSL under a gonioscope in acute PACG eyes with a mean follow-up of 20.8 months. In another study, Lai et al. [16] reported a success rate (IOP < 21 mmHg without medications) of 100% for chronic PACG patients with total PAS before surgery and the mean follow-up time was 8.9 months. In this study, with the use of an ophthalmic endoscope, our patients were followed for a longer duration of 16.4 months, with a success rate of 97.4%. So far, no study concerning GSL that compared the assistance of an endoscope and that of a gonioscope has been conducted. Theoretically, an endoscope can help to achieve a complete 360° angle open; however, whether the combined use of an endoscope in phaco-GSL leads to lower IOP than using gonioscope-assisted phaco-GSL needs further investigation.

Previous studies have shown that removal of the lens may decrease IOP in some patients with chronic PACG [17]. The mean extent of PAS was reduced from 266.41° to 198.91° by phacoemulsification alone in PACG [18]. The mechanism may be due to the mechanical deepening of the anterior chamber with viscoelastic and saline infusion during phacoemulsification, which opened PAS partially. However, another study showed that PAS was not relieved despite the dramatic deepening of the anterior chamber after phacoemulsification for chronic PACG [16]. In our study, we firstly separated the PAS using a viscoelastic agent, which could decrease the PAS range from 184.7° to 102.9°, and PAS was totally eliminated in 23.7% patients. Most cases required further mechanical separation to achieve a complete opened angle.

An unsuccessful case was present in this study, in which no pupillary block factors were involved. Although the PAS recurrence was only 90°, the trabecular pigment was grade III at 6 months postoperatively and the IOP was still not under control after glaucoma medications. Ahmed drainage valve implantation was therefore performed. The reason for the uncontrollable IOP may be caused by the occluded trabecular meshwork due to pigmentation or degeneration. Although 3/4 of the functional trabecular meshwork was exposed, the IOP could only be weakly controlled.

The long-term surgical outcome of GSL depends on the elimination of PAS-related factors. If these factors still exist, PAS will recur [16]. In the present study, 26 (68.4%) eyes suffered different degrees of PAS recurrence, ranging from 15° to 270°, but only one patient had an IOP above 21 mmHg and needed further treatment. Kameda et al. [6] showed the risk factors for postoperative failure of GSL in PACG patients including young patients and a lack of laser peripheral iridoplasty. Laser peripheral iridoplasty after GSL has been shown to make the peripheral iris flatter and reduce the probability of angle closure [16]. None of the patients were treated with laser peripheral iridoplasty in our study. In future follow-up studies, we will try to perform laser peripheral iridoplasty and study its effect of treatment.

The time interval between acute onset of PACG and surgery is an important factor that affects the postoperative IOP [19]. The trabecular meshwork may be irreversibly damaged in PACG patients who have experienced long-term angle closure [20, 21]. As a result, it is hard to achieve a satisfactory prognosis for patients who have suffered PAS for more than 1 year if GSL was not combined with phacoemulsification [20]. In our study, the mean disease duration was 38.9 months, and the longest one was up to 7 years. However, most patients got ideal IOPs, even in those who had suffered PAS for a long time. Our results are consistent with those of Zhang et al. [5], who found that 10 glaucoma patients (12 eyes) with PACG and who suffered in PAS for more than 1 year could also achieve controlled IOP after GSL. We believe that GSL is still worth trying for patients with long-term angle closure, since there are no sufficient animal or human studies to determine how much the trabecular meshwork function would recover after PAS was reopened.

Phaco-OE-GSL is also safe for treating PACG patients with cataract. The reported mean ECD loss was 5.3–17.2% in the simple phacoemulsification of a senile cataract [22–24]. The decrease in ECD was 11.54% postoperatively in our study, which was comparable with the previous reports of phacoemulsification. In our study, the most common postoperative complication was transiently elevated IOP, with an average duration of 2.7 days. The elevated IOP may be due to clogging of the trabecular meshwork by a residual viscoelastic agent or debris, or edema of the trabecular meshwork caused by the surgery. Other common postoperative complications in the present study were hyphema and anterior chamber exudation, which was consistent with the report of Fakhraie et al. [19]. In addition, 40% (6 of 15 eyes) of the patients were diagnosed with supraciliochoroidal fluid by AS-OCT. This may be related to the increased vascular permeability after surgery or a suddenly decreased IOP during the perioperative period [13, 25]. In our patients, there were no serious complications such as choroidal hemorrhage or hypotony.

GSL is usually performed by the aid of a gonioscope under an operating microscope. There are multiple disadvantages. Firstly, the direct gonioscope requires the microscope to be tilted and eyeball rotation with bridle sutures due to the oblique illumination. These inconvenient procedures make the surgical manipulation difficult. An indirect double-mirror gonioscope provides an inverse image. Although the head of the patient is not needed to be tilted, the indirect double-mirror gonioscope cannot be used in the manipulation of GSL because the edge of the lens blocks the view of corneal incision. It is used only for angle observation after GSL under an operating microscope [15]. Secondly, a blurred image is caused by corneal edema, especially in the incisional position [11]. Last but not the least, the view of the chamber angle is not clear enough under some circumstances, and repetitive GSL leads to a more obvious inflammatory reaction. In contrast, no tilting of the microscope or bridle sutures is needed during the operation with GSL by an ophthalmic endoscope. This method provides a clearer and larger site of view of the angle structures regardless of the opacity of the cornea, thus shortening the surgical time and reducing the inflammatory reaction induced by repetitive iris contact [11]. However, the high cost of the endoscope itself and the light fiber restricts the extensive use of the endoscope. Further research on cost and social benefits is therefore required.

There are limitations to this study. Firstly, a small number of patients with bilateral PACG were included in this study, which might lead to statistical bias. Also, we did not have a control group with other surgical methods; therefore, we could only compare our results with literature values. Finally, the subjects included were only Chinese, which limited the conclusions applicable to other ethnic groups.

In summary, phaco-OE-GSL is an effective and safe treatment method for chronic PACG patients with cataract. It can reopen the synechial angle to reduce IOP, and it improves visual acuity. Although a high proportion of patients experienced a postoperative recurrence of PAS, the majority could retain a satisfactory IOP level.
